# Systematic genome assessment of B-vitamin biosynthesis suggests co-operation among gut microbes

**DOI:** 10.3389/fgene.2015.00148

**Published:** 2015-04-20

**Authors:** Stefanía Magnúsdóttir, Dmitry Ravcheev, Valérie de Crécy-Lagard, Ines Thiele

**Affiliations:** ^1^Luxembourg Centre for Systems Biomedicine, University of LuxembourgEsch-sur-Alzette, Luxembourg; ^2^Department of Microbiology and Cell Science, Institute of Food and Agricultural Sciences and Genetics Institute, University of FloridaGainesville, FL, USA

**Keywords:** B-vitamin biosynthesis, gut microbiota, genome annotation, PubSEED, subsystem

## Abstract

The human gut microbiota supplies its host with essential nutrients, including B-vitamins. Using the PubSEED platform, we systematically assessed the genomes of 256 common human gut bacteria for the presence of biosynthesis pathways for eight B-vitamins: biotin, cobalamin, folate, niacin, pantothenate, pyridoxine, riboflavin, and thiamin. On the basis of the presence and absence of genome annotations, we predicted that each of the eight vitamins was produced by 40–65% of the 256 human gut microbes. The distribution of synthesis pathways was diverse; some genomes had all eight biosynthesis pathways, whereas others contained no de novo synthesis pathways. We compared our predictions to experimental data from 16 organisms and found 88% of our predictions to be in agreement with published data. In addition, we identified several pairs of organisms whose vitamin synthesis pathway pattern complemented those of other organisms. This analysis suggests that human gut bacteria actively exchange B-vitamins among each other, thereby enabling the survival of organisms that do not synthesize any of these essential cofactors. This result indicates the co-evolution of the gut microbes in the human gut environment. Our work presents the first comprehensive assessment of the B-vitamin synthesis capabilities of the human gut microbiota. We propose that in addition to diet, the gut microbiota is an important source of B-vitamins, and that changes in the gut microbiota composition can severely affect our dietary B-vitamin requirements.

## Introduction

The human gut microbiota (HGM) supplies its host with several nutrients, including amino acids (Metges, 2000) and certain B-vitamins (Hill, 1997; Rossi et al., 2011). B-vitamins are necessary cofactors for numerous aspects of human metabolism, including fat and carbohydrate metabolism and DNA synthesis. Human cells are not capable of producing B-vitamins in sufficient amounts; thus, these cells must obtain such vitamins either from the human diet or the gut microbiota (Rucker et al., 2007).

B-vitamins are found in many food products, but they are water-soluble and many of them are temperature sensitive; thus, these vitamins can easily be removed or destroyed during the cooking process. B-vitamin deficiency is common in humans and supplements of these vitamins are often used because deficiencies in these vitamins can lead to several diseases, such as pellagra (Rucker et al., 2007). Therefore, there is great interest in the B-vitamin production by the gut microbiota, particularly because this production is thought to play a role in maintaining the vitamin homeostasis in colonocytes (Rucker et al., 2007). Because of the large number of gut microbial species, most of which have not been cultured, experimental validation of the HGM B-vitamin pathways is infeasible, at least with the current technologies. Genome annotations provide a method for both systematic and large-scale predictions of pathways for vitamin metabolism, enabling assessments of the potential of each species for vitamin biosynthesis.

Genome annotations can be obtained using, among other methods, a genomics-based approach implemented in the PubSEED platform (Overbeek et al., 2005; Disz et al., 2010). PubSEED is a multifunctional web platform, in which one can inspect defined sets of gene functional roles (annotations) for multiple genomes at once and assign annotations to protein encoding genes (PEGs), which refer to open reading frames (ORFs). A functional role can refer to any protein-encoding gene and represents the protein function. Communities of experts define sets of functional roles, called subsystems. These subsystems usually contain functional roles that together form a metabolic pathway for a particular subset of metabolism, for example, the biosynthesis of vitamin B1 (thiamin). The subsystem can then be annotated over any number of genomes at once. In addition, the PubSEED platform offers multiple methods for the manual curation, discovery, and assignment of functional roles.

In this study, we used the PubSEED platform to analyze 256 HGM organisms for their ability to synthesize eight B-vitamins. The genomes were selected based on a published collection of microorganisms commonly found in the human gut (Qin et al., 2010). We used eight B-vitamin subsystems, which correspond to the biosynthesis of the active form of each vitamin from its known precursors, to predict the possible B-vitamin producers in the human gut as well as the potential competitors for resources from the vitamin pools in the gut. In addition, we compared our predictions to experimental data available in the literature on the B-vitamin requirements of 16 human gut microorganisms. This work presents a comprehensive assessment of the B-vitamin synthesis capabilities of the human gut microbiota.

## Materials and methods

The PubSEED platform (http://pubseed.theseed.org/) (Overbeek et al., 2005, 2013; Disz et al., 2010) contains fully annotated subsystems for eight B-vitamins: biotin, cobalamin, folate, niacin, pantothenate, pyridoxine, riboflavin, and thiamin (Table 1). The subsystems were populated with 256 HGM genomes. The genomes were selected based on a study by Qin et al. (2010), which reported microorganisms commonly found in the human gut. A further selection criterion was that the microbes appeared in at least 50% of the participants in the aforementioned study and that their genomes were present in PubSEED (Supplementary Table 1). For comparison, the subsystems were also populated with 257 non-HGM genomes that were isolated from human body sites other than the intestine (Supplementary Table 1). The non-HGM genomes were chosen in the following way: (1) All genomes from the Human Microbiome Project (HMP, http://www.hmpdacc.org/HMRGD/) (The Human Microbiome Project Consortium, 2012a,b) isolated from a human body site were selected; (2) HMP genomes with the body site “Gastrointestinal tract” (i.e., isolated from intestine) were excluded; (3) all genomes absent from PubSEED were excluded; (4) only one genome per species was selected; (5) among the multiple genomes for the same species, a genome with a minimal number of contigs (i.e., definitive) was selected; (6) if more than one genome with the minimal number of contigs existed, the genome with the maximal CDS number was selected. The non-HGM genomes were isolated from the following body sites: oral (127 genomes), urogenital tract (68 genomes), airways (28 genomes), skin (25 genomes), blood (7 genomes), liver (1 genome), and heart (1 genome).

**Table 1:** **Subsystems analyzed for the studied B-vitamins**.

**Vitamin**	**Subsystem name**	**Subsystem curator**
Biotin	“Biotin biosynthesis”	Dmitry Rodionov
Cobalamin	“Coenzyme B12 biosynthesis”	Dmitry Rodionov
Folate	“Folate Biosynthesis”	Valérie de Crécy-Lagard
Niacin	“NAD and NADP cofactor biosynthesis global”	Andrei Osterman
Pantothenate	“Coenzyme A Biosynthesis”	Andrei Osterman
Pyridoxine	“Pyridoxin (Vitamin B6) Biosynthesis”	Olga Zagnitko
Riboflavin	“Riboflavin, FMN, and FAD metabolism Extended”	Sveta Gerdes
Thiamin	“Thiamin biosynthesis”	Dmitry Rodionov

The subsystems can be accessed on the PubSEED Subsystems Editor website: http://pubseed.theseed.org/

### Preparing and populating the subsystems

We first inspected the existing B-vitamin metabolism subsystems and determined whether the functional roles associated with the metabolism of each vitamin were consistent with most of the recent reports. To enable us to manually curate the functional roles involved in the biosynthesis of each vitamin, we copied the existing subsystems to new subsystems. We then compiled a list of HGM genomes by selecting a genome in the “Spreadsheet” tab and clicking “save genome selection.” Using “Edit List,” we selected our genomes and saved the list. Within the spreadsheet for each subsystem, we limited the display of genomes to our genome list in the window “User Sets.” To simplify our analysis, we compiled functional roles with the same metabolic functions into subsets using the “Subsets” tab. Protein encoding genes (PEGs), which encode functional roles belonging to a certain pathway, often cluster together on a chromosome. In the tab “Color Spreadsheet,” we colored the genes by cluster to better identify these co-occurring genes. Finally, for each B-vitamin subsystem, we determined sets of essential functional roles that should be present in a genome for the corresponding organism to be considered a vitamin producer (Table 2). Missing essential roles in HGM genomes were manually curated where possible as described below. The 257 non-HGM genomes were subjected to the same criteria as those for the 256 HGM genomes.

**Table 2:** **Combinations of essential functional roles for the biosynthesis the B-vitamins**.

**Pathway**	**Essential functional roles**
Biotin	BioW + BioFADB
	BioC + BioFADB
Cobalamin	CbiL + CobG + CbiGF + (CobF or CbiD) + CbiECA + CobNST+ CobAT + CbiPB + CobUS
	(CbiKX or CysG) + CbiLGF + (CobF or CbiD) + CbiECA + CobAT + CbiPB + CobUS
Folate	FolEBKP + pabAc + DHFS + DHFR + FPGS
Niacin	ASPOX + QSYN + QAPRT + NaMNAT + NADS
Pantothenate	KPHMT + KPRED + ASPDC + PBAL + PANK + PPCS + PPCDC + DPCK
Pyridoxine	dxs + gapA + PdxBFAJH
	PdxTS
Riboflavin	GTPCH2 + PyrDR + DHBPS + DMRLS + RSA + RK + FMNAT
Thiamin	(ThiH or ThiO) + ThiGSF + ThiCDE
	Thi4 + ThiCDE

*When more than one biosynthetic pathway for one vitamin is possible, several combinations of the essential roles are listed. Each abbreviation corresponds to the single functional role (Supplementary Table 2)*.

### Manual curation

The PubSEED platform offers several methods for manual curation. The function “Find candidates” uses four steps to search for a functional role in a target genome. First, it looks for the role in the target genome's existing gene annotations. Then, it searches for matching proteins in the genome by performing a similarity check with protein BLAST algorithm (Altschul et al., 1990), where it compares the amino acid sequence of the gene from another genome with the amino acid sequences of all PEGs in the target genome. The third step searches for genes, which are co-localized on the chromosome with other genes from the subsystem in the target genome. Finally, a translated BLAST (tblastn) is performed, in which a query protein sequence is compared with the six-frame translation of the target genome. Another way to search for functional roles in the PubSEED platform is to examine genome annotation phylogenetic trees for sets of related proteins. Such trees show similarities among the amino acid sequences of proteins from different genome clusters together with the annotated functional role of each PEG. Trees often reveal mis-annotations of the target genes and also provide hints of the level of conservation of the sequence. For every protein-encoding gene in the PubSEED platform, it is possible to examine the NCBI Conserved Domains Database (CDD) (Marchler-Bauer et al., 2013). The database performs an RPS-BLAST algorithm (a stand-alone tool in the NCBI toolkit distribution), presents the resulting protein domain annotations on the query sequence, and lists the resulting *E*-values. High-confidence matches between the query sequence and conserved domains are listed as “specific hits.” Once a candidate gene was found in a genome, the functional role of the PEG was changed manually on the condition that the existing annotation was not associated with another subsystem, because changing the annotation of a PEG affects all subsystems that list the current annotation can alter the results of an existing subsystem without notifying its author. Either a new functional role was assigned to the PEG, or in the case of multiple domains or functions, the new annotation was appended to the existing ones.

### Calculation of vitamin produced by gut microbes

We calculated the minimal number of microbes needed to supply a human host with the necessary B-vitamins using the following assumptions: (i) The number of bacterial cells in the colonic space is 10^14^ cells (Savage, 1977). (ii) The dry weight of a single bacterial cell is 4.89 ^*^ 10^−13^ gDW cell^−1^ (Loferer-Krößbacher et al., 1998). (iii) Intracellular vitamins in bacteria become available to the host upon cell lysis. (iv) 31.7% of bacterial cells in fecal matter has been reported to consist of dead cells (Ben-Amor et al., 2005). The ratio of dead cells in the colon could not be found, and we therefore use the measured ratio of dead cells in the feces in our calculations. (v)The intracellular concentration of vitamins was retrieved from the literature (Table 3) but represents only the capacity of those bacteria and not all gut bacteria in general. (vi) The volume of an *E. coli* cell is 1.1 μm^3^ (Kubitschek and Friske, 1986). (vii) The dry weight of an *E. coli* cell is 4.89 ^*^ 10^−13^ gDW (Loferer-Krößbacher et al., 1998). (viii) *E. coli* grows anaerobically at 0.26 h^−1^ (Hasona et al., 2004).

**Table 3:** **Intracellular vitamin concentrations in selected gut microbes**.

**Vitamin**	**Organism**	**Reported value**	**References**
Biotin	*E. coli*	40 μM	Cicmanec and Lichstein, 1978
Cobalamin	*Lactobacillus reuteri* CE	50 μg/L	Taranto et al., 2003
Flavin	*E. coli*	4 μM	Wilson and Pardee, 1962
Folate derivatives	*Lactobacillus casei*	22 μM	Shane and Stokstad, 1976
Niacin	*E. coli*	1.12 ^*^ 10^6^ molecules in a single cell	McLaren et al., 1973
Pantothenate	*E. coli*	<1 μM	Jackowski and Alix, 1990
Pyridoxine	*E. coli*	1.5 ^*^ 10^−10^ mol h^−1^ mgDW^−1^	Dempsey, 1971
Thiamine	*Lactobacillus fermenti*	3 μg/gDW	Sompolinsky and Neujahr, 1971

The ratio of the human daily reference intake of each vitamin coming from bacteria could then be calculated in the following manner:

$$\%\;of\;DRI=\frac{\begin{array}{l} Intracellular\;concentration\;*Weight\;of\\ bacteria\;*\;AM\;*\;HGM\;ratio \end{array}}{DRI}*\;100$$

where DRI is the recommended dietary reference intake in mg/day, AM is the atomic mass of the vitamin [values from ChEBI, (Hastings et al., 2013)], and HGM is the ratio of HGM producers as predicted in this study.

## Results

### Pathway descriptions, prediction criteria, and predictions

For each of the eight B-vitamins, we describe the known biosynthesis pathways and for each pathway, we present the frequency of the respective functional roles in the analyzed taxonomic groups. Based on the functional roles present in the subsystems, each HGM organism was predicted to be a producer or non-producer of the eight B-vitamins (Supplementary Table 1).

#### Biotin (Vitamin B8)

**Figure 1:**
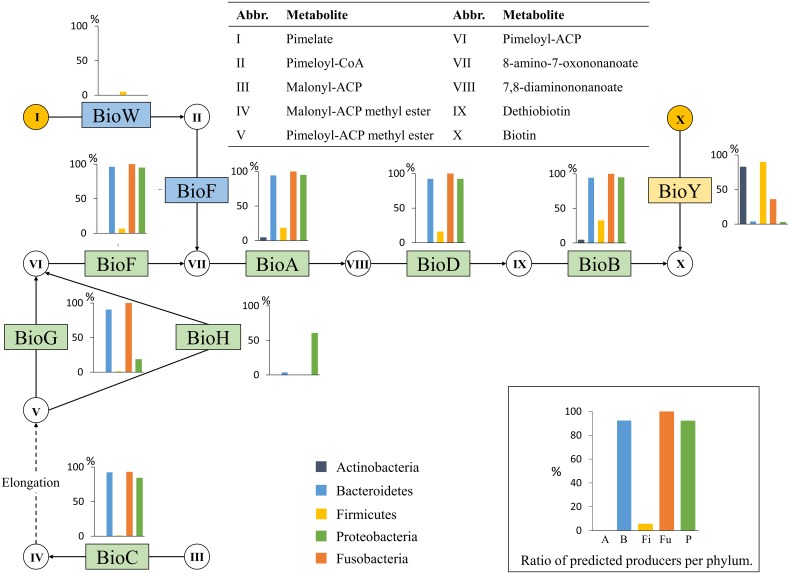
**Biotin biosynthesis**. The biotin biosynthesis subsystem contains 9 functional roles (Supplementary Table 2) and 10 metabolites. Functional roles are represented by rectangles. The major biosynthesis routes are colored green, alternative routes are blue, salvage routes are red, and known transporters are yellow. Metabolites are represented by circles, and yellow circles represent metabolites that can be salvaged from the environment in some cases. Only the core metabolites of the biosynthesis pathway are listed. Each bar graph represents the percentage of organisms in each phylum that contain the functional role.

Biotin can be synthesized *de novo* from two pimeloyl precursors, malonyl-ACP and pimelate (Figure 1). There are five different synthesis routes (Rodionov et al., 2002; Lin and Cronan, 2011) but only three, via BioG, BioH, and BioW, could be identified in the 256 HGM genomes.

We examined the prevalence of the BioC route using the BioG role in the 256 HGM genomes. Almost all genomes from the phylum Bacteroidetes (96%) were predicted to synthesize biotin through this route. All analyzed genomes from the phylum Fusobacteria were predicted to synthesize biotin through the BioC route with BioG. A single Fusobacteria genome, *Fusobacterium* sp. D11, was missing the essential role BioC, but it was still predicted to be a producer because the genome contained all other essential roles and all its related genomes contained BioC. In the phylum Proteobacteria, 84% of the genomes were predicted to synthesize biotin. Producers from the class Epsilonproteobacteria contained the BioG role, whereas the remaining producers in the phylum, which were mostly from the class Gammaproteobacteria, contained a BioH. Only a single Gammaproteobacteria, *Escherichia* sp. 1_1_43, was a predicted non-producer.

We then examined the second biosynthesis route, in which BioW is used to convert salvaged pimelate to pimeloyl-CoA. The BioFADB pathway is then used to convert pimeloyl-CoA to biotin. This route was only observed in the Firmicutes phylum in five genomes from the Clostridia class and in *Bacillus subtilis* subsp. *subtilis* str. 168. During our analysis, we observed that all Actinobacteria genomes lacked the essential biotin biosynthesis roles. However, 19 of the 23 (83%) Actinobacteria genomes contained a BioY biotin transporter, indicating a need for biotin. The remaining four genomes, all from the family Coriobacteriaceae, contained no functional roles in the pathway at all, but they did contain biotin-protein ligases, suggesting that they require biotin.

Taken together, these findings show that the majority of Bacteroidetes, Fusobacteria, and Proteobacteria genomes contain the essential roles for biotin biosynthesis (Figure 1). The synthesis of this vitamin is completely absent in Actinobacteria genomes and is very rare in genomes of the Firmicutes phylum. In total, biotin biosynthesis is present in 40% of the HGM genomes.

#### Cobalamin (Vitamin B12)

**Figure 2:**
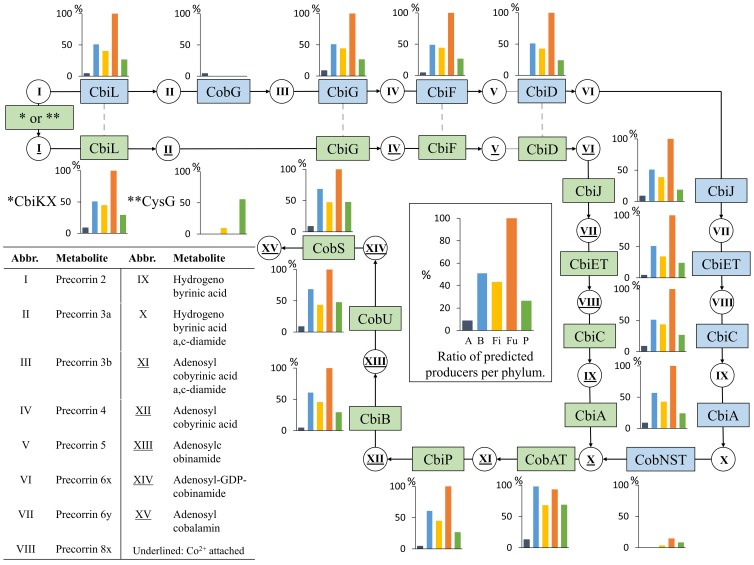
**Cobalamin biosynthesis**. The cobalamin biosynthesis subsystem contains 17 functional roles (Supplementary Table 2) and 15 metabolites. Refer to Figure 1 for figure descriptions. Note that we show only CbiD since none of the HGM genomes contained CobF. The “^*^ or ^**^” text in the first green box on the top left corner of the figure refer to two functional roles “^*^CbiKX” and “^**^CysG”. The corresponding bar charts for the two functional roles are shown below the green box, just above the “Abbr./Metabolites” table.

Cobalamin biosynthesis contains the longest pathway of eight vitamins, where adenosylcobalamin is synthesized from precorrin 2 (Figure 2). The cobalamin can be synthesized either aerobically or anaerobically (blue and green pathways in Figure 2, respectively).

No HGM microbe used the aerobic biosynthesis route, whereas some non-HGM producers contained the aerobic pathway specific roles CobG and CobNST. Only two Actinobacteria of the Coriobacteriaceae family were predicted cobalamin producers, *Collinsella aerofaciens* ATCC 25986 and *Gordonibacter pamelaeae* 7-10-1-b. Only half of the genomes in the Bacteroidetes phylum (26 of 51) were predicted to be a cobalamin producers being the lowest producer ratio observed for the Bacteroidetes of all eight vitamins. No taxonomic patterns were observed for the Bacteroidetes producers. Nearly half of the Firmicutes genomes (43%) were predicted to synthesize cobalamin. All Lactobacillales were predicted to be non-producers, except the six *Lactobacillus reuteri* strains that have been previously known to have this pathway (Santos et al., 2008). Only a single other Bacilli, *Listeria monocytogenes* str. 1/2a F6854, was a predicted producer of this vitamin. The remaining cobalamin producers in the Firmicutes phylum belonged to the Clostridia class, but no specific taxonomic pattern was observed. All 14 Fusobacteria were predicted to be cobalamin producers. The Proteobacteria phylum contained only ten producers, three from Delta- and seven from Gammaproteobacteria.

Taken together, we find that cobalamin biosynthesis is present in 42% of the HGM genomes. The synthesis of cobalamin is present in all Fusobacteria, but is rare in Actinobacteria and Proteobacteria. Half of the Bacteroidetes genomes are missing the biosynthesis pathway, making cobalamin the least produced of the eight vitamins in the phylum.

#### Folate (Vitamin B9)

**Figure 3:**
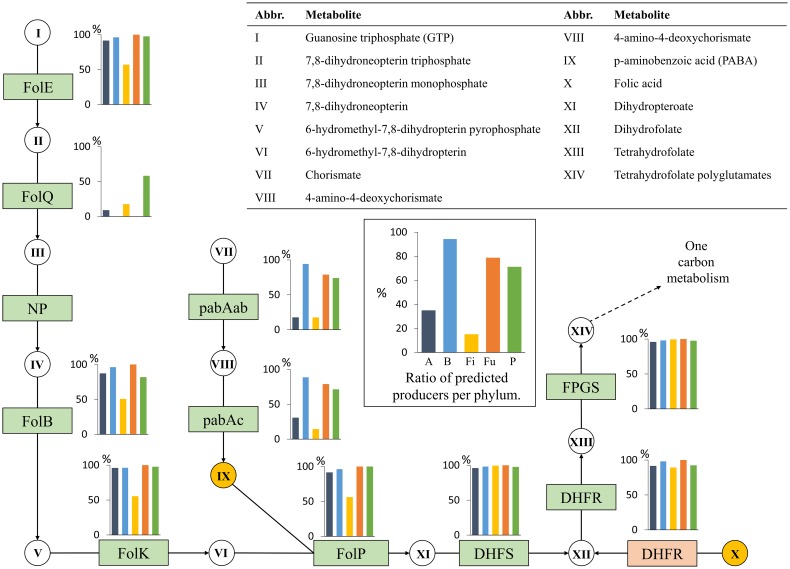
**Folate biosynthesis**. The folate biosynthesis subsystem contains 11 functional roles (Supplementary Table 2) and 14 metabolites. Refer to Figure 1 for figure descriptions.

Folate biosynthesis includes the combination of two metabolic branches (Figure 3). The first branch converts guanosine triphosphate (GTP) to 6-hydromethyl-7,8-dihydropterin and the second branch converts chorismate to *p*-aminobenzoic acid (PABA). Most living organisms contain more than one folate derivative. However, because these folates are derived from dihydrofolate (DHF) or tetrahydrofolate (THF), we considered the production of either metabolite to be sufficient to assign folate producer status to a genome.

From our data, we identified 109 predicted folate producers, 86% of which included all the functional roles of the pathway or were only missing the FolQ role, which is commonly missing in genome annotations (Gerdes et al., 2012). All producers were required to have the pabAc role of the PABA synthesis branch of the pathway. The two other roles, pabAa and pabAb, were commonly missing from our genomes, and the assignment of the roles from manual inspection was not trivial. Therefore, the presence of pabAa or pabAb was not considered essential, although these roles, along with pabAc, are needed for the synthesis of PABA. A PABA auxotroph must acquire the metabolite from its growth medium and was therefore not considered to be capable of producing folates *de novo*. Only six (26%) Actinobacteria were predicted producers: five from the order Bifidobacteriales and one from the order Actinomycetales. Although most genomes in this phylum had all the necessary functional roles for THF production, they did not contain pabAc. All but four (92%) Bacteroidetes were predicted to synthesize folates; the four exceptions lacked pabAc. Only 18 Firmicutes had complete synthesis pathways. Of the remaining 112 Firmicutes genomes, 49 contained a full biosynthesis pathway but were missing the PABA branch. The other genomes were predicted non-producers, but contained roles for the conversion of dihydropteroate to THF, suggesting that they rely on folate uptake. Three Fusobacteria were predicted to be PABA auxotrophs, whereas the remaining 11 (79%) genomes contained all the essential roles for folate biosynthesis. The Proteobacteria phylum demonstrated more variability and class-specific patterns, with 71% of the organisms predicted as producers. The Betaproteobacteria included three producers, whereas two genomes from this class were missing PABA biosynthesis. All Deltaproteobacteria were predicted to be non-producers. The Gammaproteobacteria demonstrated the highest conservation of the pathway; all functional roles were present. Only two non-producers were observed in the class: *Succinatimonas hippei* YIT 12066 was missing the PABA branch, and *Escherichia* sp. 1_1_43 was missing the PABA branch, FolE, DHFS, and FPGS.

Taken together, these findings show that the folate biosynthesis pathway is present in nearly all Bacteroidetes genomes as well as in most Fusobacteria and Proteobacteria. Folate synthesis is rare in the Actinobacteria and Firmicutes genomes, mostly because of the absence of the PABA biosynthesis pathway. In total, folate biosynthesis is complete in 43% of the 256 HGM genomes tested.

#### Niacin (Vitamin B3)

**Figure 4:**
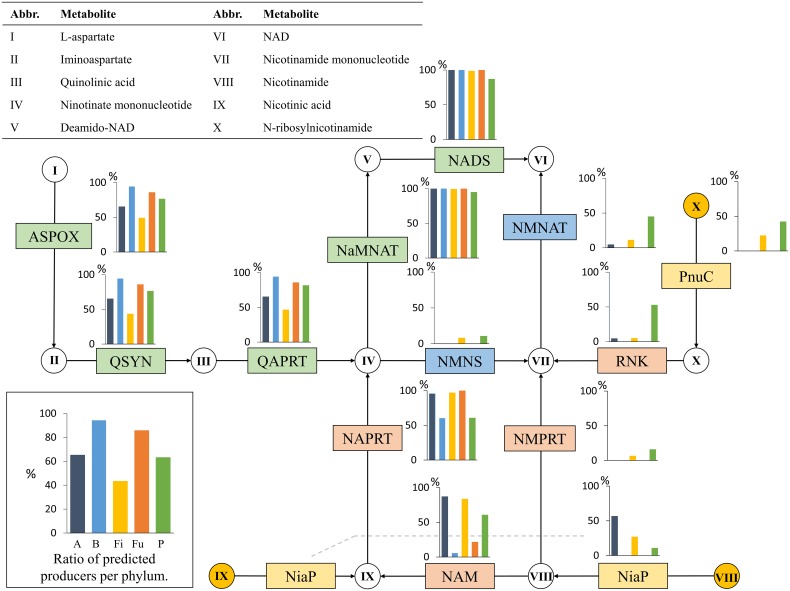
**Niacin biosynthesis**. The niacin biosynthesis subsystem contains 14 functional roles (Supplementary Table 2) and 10 metabolites. Refer to Figure 1 for figure descriptions.

Niacin is a group term for nicotinamide and nicotinic acid, and both of these metabolites are precursors for nicotinamide adenine dinucleotide (NAD). Nicotinamide and nicotinic acid can either be salvaged from the environment or produced through the recycling of NAD within the cell (Gossmann et al., 2012). In this study, an organism was considered a niacin producer when it contained the *de novo* synthesis pathway of NAD (Begley et al., 2001) (Figure 4).

The first route uses the functional role NaMNAT to produce deamino-NAD, which in turn is converted to NAD by NADS. NADS requires ammonia, or alternatively, NADS is coupled to a glutaminase domain (GAT) that supplies ammonia (De Ingeniis et al., 2012). In this study, the presence or absence of the GAT domain did not affect our predictions. All predicted producers among the 256 genomes contained both NaMNAT and NADS. In the case of Firmicutes, the Bacilli class contained only five producers and the Clostridia class included 44 predicted niacin producers. Most Fusobacteria were predicted to synthesize niacin, with only two predicted non-producers. Proteobacteria contained 29 predicted niacin synthesizers, but no taxa-specific patterns were observed.

Considering the prevalence of the first biosynthesis route in the gut microbiota, we wondered how common the alternative synthesis route was in these genomes. This route produces nicotinate D-ribonucleotide from quinolinic acid through NMNS and converts it to NAD using NMNAT. Only eight of the analyzed genomes contained both of these functional roles, and all contained the roles NaMNAT and NADS from the first route as well. All eight genomes belonged to the Firmicutes phylum, seven from the Clostridia class and one from Erysipelotrichia. Some prokaryotic cells share the eukaryotic NAD biosynthesis pathway from tryptophan (Kurnasov et al., 2003). However, we did not observe any evidence of an active tryptophan pathway in our list of HGM genomes. Because niacin is known to be salvaged from the environment, we investigated the functional roles associated with the uptake of three NAD precursors: nicotinamide, nicotinic acid, and N-ribosylnicotinamide. We found that the roles associated with the uptake of the nicotinamide and nicotinic acid (Figure 4) were only present in Actinobacteria, Firmicutes, and a single Proteobacteria. Two roles associated with the conversion of salvaged niacin to nicotinate mononucleotide (NAM and NAPRT) were present in all five phyla, but these roles are involved in the recycling of NAD (Gossmann et al., 2012) and therefore do not necessarily indicate the presence of niacin salvage. The salvage of N-ribosylnicotinamide through PnuC, RNK, and NMNAT was only present in four Firmicutes and 18 Proteobacteria.

Taken together, these findings show that niacin biosynthesis is present in the majority of the HGM genomes. The phyla Actinobacteria and Firmicutes contain lower ratios of *de novo* producers than the other three, and in these phyla, we observed the presence of the niacin salvage pathways, which are not present in the Fusobacteria and Bacteroidetes genomes. A total of 63% of all the investigated genomes contained the NAD biosynthesis pathways.

#### Pantothenate (Vitamin B5)

**Figure 5:**
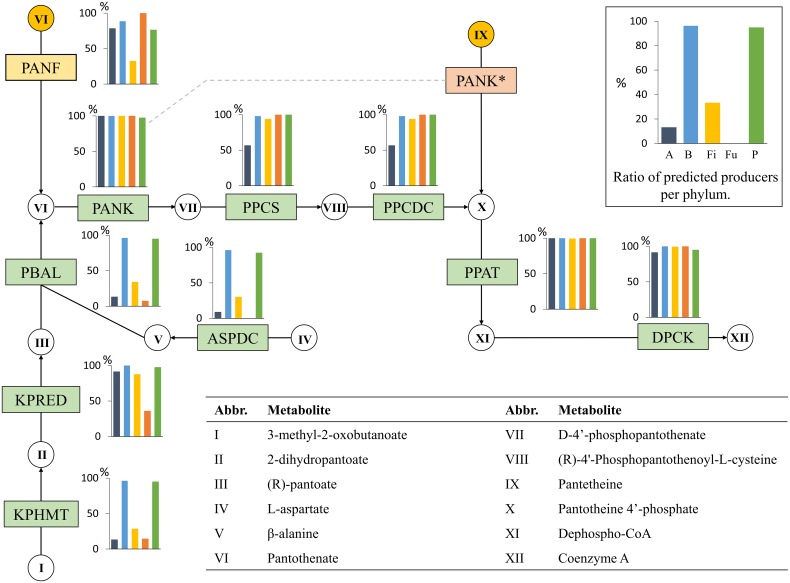
**Pantothenate biosynthesis**. The pantothenate biosynthesis subsystem contains 10 functional roles (Supplementary Table 2) and 12 metabolites. Refer to Figure 1 for figure descriptions.

Pantothenate is a precursor for coenzyme A (CoA) and it can be synthesized *de novo* from 2-dihydropantoate and β-alanine (Figure 5). Because CoA is the active form of pantothenate, we considered the *de novo* pantothenate biosynthesis pathway to be complete when a genome contained the functional roles needed for CoA biosynthesis. Although this pathway is well defined, it caused many uncertainties in our analysis. Many of the analyzed genomes were missing the roles KPHMT or ASPDC. When a genome was missing a single enzyme in the branched pathway, the organisms were still predicted to be a pantothenate producer. Some genomes were lacking the role PBAL, which is the final step of pantothenate biosynthesis. Hence, the absence of PBAL always resulted in a non-producer prediction.

According to our predictions, Fusobacteria, certain Actinobacteria, and Firmicutes do not possess the ability to synthesize pantothenate (Figure 5). These predictions were mostly based on the absence of two or more of the functional roles KPHMT, KPRED, ASPDC, and PBAL. All Bacteroidetes and 36 (95%) of the Proteobacteria genomes were predicted to be CoA producers. Only three Actinobacteria were predicted CoA producers: two of the Actinomycetales order and one Coriobacteriales. None of the Bifidobacteriales contained a full CoA biosynthesis pathway, but all genomes in the order contained the pantothenate transporter PANF. The four non-producing Coriobacteriales genomes did not contain a PANF transporter. Firmicutes displayed certain class-level similarities, with 32% of the genomes containing the essential roles for CoA biosynthesis. Only seven Bacilli, including three Bacillales and four Lactobacillales, contained all CoA-biosynthesis roles. The Clostridia class demonstrated considerable variability in the presence of roles, and no specific pattern could be observed for the lower taxa. In the phylum Proteobacteria, all organisms of the Betaproteobacteria class except one contained all essential functional roles for CoA biosynthesis, whereas none of the producers presented a PANF transporter. All Delta- and Epsilonproteobacteria were predicted to be producers. All Gammaproteobacteria contained all roles in the pathway, except a single genome of the order Aeromonadales was missing three out of the four necessary steps for pantothenate biosynthesis.

Taken together, these findings demonstrate that the synthesis of CoA from pantothenate is present in nearly all HGM genomes. However, pantothenate biosynthesis is not present in Fusobacteria and is only present in a few Actinobacteria and Firmicutes. Nearly all Bacteroidetes and Proteobacteria contain a full biosynthesis pathway for both pantothenate and CoA. CoA biosynthesis is present in 51% of the HGM genomes.

#### Pyridoxine (Vitamin B7)

**Figure 6:**
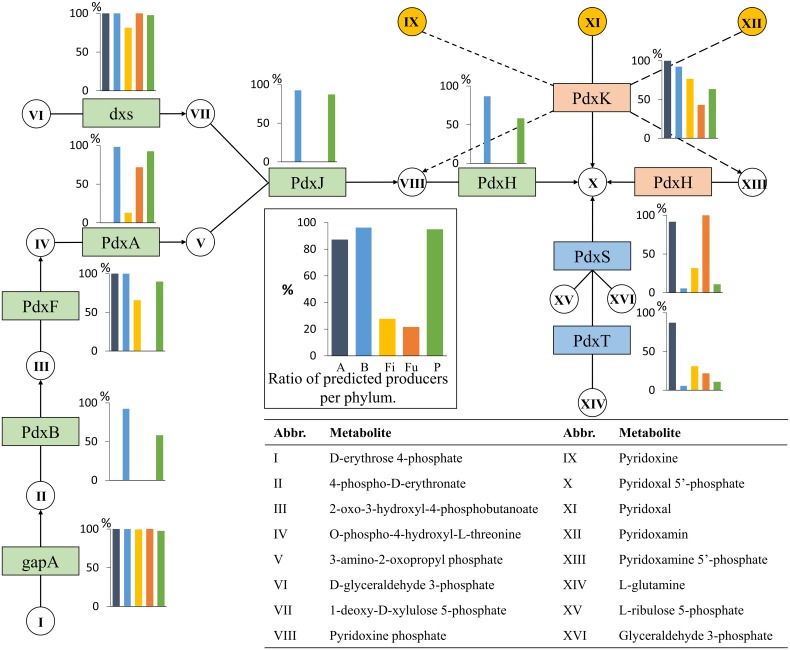
**Pyridoxine biosynthesis**. The pyridoxine biosynthesis subsystem contains 11 functional roles (Supplementary Table 2) and 16 metabolites. Refer to Figure 1 for figure descriptions.

The coenzyme form of pyridoxine is pyridoxal 5′-phosphate and is required for several enzymes in the cell, mainly those required for the metabolism of amino acids (Dakshinamurti and Dakshinamurti, 2007). Pyridoxal 5'-phosphate can be synthesized *de novo* via two different routes (Figure 6).

We first analyzed the shorter biosynthesis route, which uses a single enzyme with two domains, represented by the functional roles PdxS and PdxT. This route joins glyceraldehyde 3-phosphate and D-ribulose 5-phosphate to produce pyridoxal 5′-phosphate. Both roles must be present for an organism to be a predicted producer. The 20 Actinobacteria and 33 Firmicutes genomes that were predicted to be pyridoxine producers contained the PdxTS roles. Only three Bacteroidetes producers contained the PdxTS route: two from the Prevotellaceae family and one Rikenellaceae, *A. indistinctus* YIT 12060, which also contained the alternative biosynthesis route described below. The three Fusobacteria producers contained PdxTS; four Proteobacteria producers (i.e., the three Deltaproteobacteria and a single Gammaproteobacteria, *A. junii* SH205) also contained PdxTS.

We then examined the alternative synthesis route, which requires seven functional roles for a complete pathway made of two branches. One branch converts D-erythrose-4-phosphate to 3-amino-2-oxopropyl phosphate in four steps. The other branch converts D-glyceraldehyde-3-phosphate to 1-deoxy-D-xylulose-5-phosphate with the functional role dxs (Figure 6). The two end-metabolites are joined by PdxJ and converted to pyridoxal 5′-phosphate by PdxH. Both PdxJ and PdxH were required for an organism to be a predicted pyridoxine synthesizer. Only genomes from the Bacteroidetes and Proteobacteria phyla contained the roles PdxJ and PdxH in addition to the remaining essential functional roles of the pathway (Table 2); therefore, Bacteroidetes and Proteobacteria were the only phyla predicted to synthesize pyridoxine via this route. All Gammaproteobacteria, with the exception of *Escherichia* sp. 1_1_43, contained a full PdxJH biosynthesis route. Only three Bacteroidetes genomes—*Bacteroides coprocola* DSM 17136, *Bacteroides coprophilus* DSM 18228, and *Bacteroides plebeius* DSM 17135—were predicted to be non-producers. All three genomes were missing PdxH but contained all other functional roles of the pathway.

The results for the two routes showed that the majority of Actinobacteria, Bacteroidetes, and Proteobacteria have the ability to synthesize pyridoxal 5′-phosphate. Most Bacteroidetes and Proteobacteria use the longer route, and all Actinobacteria contain the shorter synthesis route. Only few Firmicutes and Fusobacteria can synthesize pyridoxal 5′-phosphate, but those that do use the shorter PdxTS route. Taken together, these findings reveal that the *de novo* biosynthesis of pyridoxal 5′-phosphate is present in 50% of the tested HGM genomes. The majority of genomes from all phyla, except for Fusobacteria, contained the role PdxK, which is involved in the salvage of pyridoxal 5′-phosphate and its two precursors.

#### Riboflavin (Vitamin B2)

**Figure 7:**
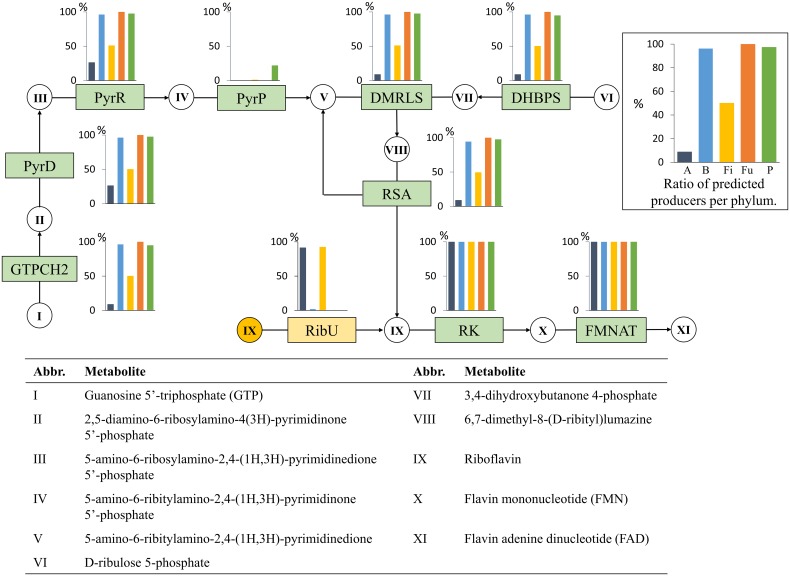
**Riboflavin biosynthesis**. The riboflavin biosynthesis subsystem contains 10 functional roles (Supplementary Table 2) and 11 metabolites. Refer to Figure 1 for figure explanations.

Riboflavin can only be synthesized through one known pathway from GTP and D-ribulose-5-phosphate (Figure 7). The role PyrP is commonly missing in our genomes and is commonly missing in plants (Gerdes et al., 2012); thus, its absence did not affect our predictions. For an organism to be considered a predicted producer, its genome had to present all other functional roles to produce the redox cofactors FMN and FAD.

All Bacteroidetes and Fusobacteria and 36 genomes (92%) of Proteobacteria contained all essential functional roles for riboflavin biosynthesis (Figure 7, Table 2). Half of the Firmicutes were predicted to be riboflavin producers. This ratio is the largest in Firmicutes producers among all of the vitamins in our analysis. Although riboflavin biosynthesis was the most prominent in Firmicutes, no taxa-specific patterns were found. Only two genomes in the Actinobacteria phylum had all the essential roles for riboflavin biosynthesis: *Corynebacterium ammoniagenes* DSM 20306 and *Bifidobacterium longum* ATCC 15697. All Bacteroidetes were predicted producers. All but two genomes from Proteobacteria were predicted to synthesize riboflavin.

The riboflavin biosynthesis pathway was the most preserved of the eight vitamins we examined, although it was absent in most of the Actinobacteria genomes and half of the Firmicutes genomes. However, all non-producing organisms from the two phyla contained the RibU riboflavin transporter role, indicating their need for the riboflavin-derived cofactors FMN and FAD. RibU was almost completely absent in the Bacteroidetes, Fusobacteria, and Proteobacteria, whereas the *de novo* synthesis pathway was found in nearly all genomes of the three phyla. The majority of all 256 genomes (65%) had the ability to produce riboflavin.

#### Thiamin (Vitamin B1)

**Figure 8:**
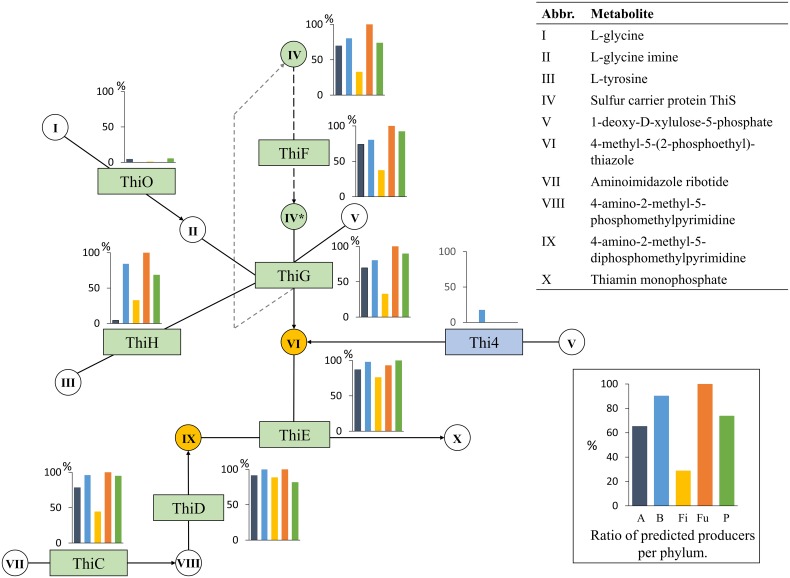
**Thiamin biosynthesis**. The thiamin biosynthesis subsystem contains 9 functional roles (Supplementary Table 2) and 10 metabolites. One functional role, sulfur carrier protein This, is drawn as a metabolite because it is combined with other metabolites and recycled in the pathway. Refer to Figure 1 for figure explanations.

The thiamin biosynthesis pathway consists of two branches that are joined in the final step to produce thiamin monophosphate (Figure 8) (Jurgenson et al., 2009).

All genomes in our analysis contained the ThiCD functional roles. The Thi4 role was only found in nine genomes, which were all from the Bacteroidetes phylum. The second HET-P synthesis route requires four functional roles: ThiS, ThiG, ThiF, and ThiO or ThiH. The roles ThiO and ThiH convert glycine and tyrosine, respectively, to glycine imine. The role ThiS carries a sulfur group to ThiG, which combines the sulfur with glycine imine and DXP to produce HET-P. Most predicted producers in our analysis used the ThiH role except for four genomes that contained ThiO (two from the Proteobacteria, one from the Actinobacteria, and one from the Firmicutes phylum). Only two Actinobacteria out of its 15 producers contained the ThiH or ThiO part of the pathway; because their genomes contained all other functional roles, they were predicted to be producers. In the final step, HET-P and HMP-PP are combined to produce thiamin monophosphate by ThiE. Although thiamin diphosphate is the functional version of thiamin, the production of thiamin monophosphate was considered sufficient for producer status. Only a single Bacteroidetes genome, *Prevotella salivae* DSM 15606, was missing essential functional roles for thiamin biosynthesis. In the Firmicutes phylum, no Bacilli received a producer status, whereas half of the Clostridia class genomes were predicted to synthesize thiamin. In Proteobacteria, all but one Gammaproteobacteria genome were predicted to be producers. The remaining seven producers from the phylum belonged to the Beta-, Delta-, and Epsilonproteobacteria classes.

Taken together, these results show that the synthesis of thiamin monophosphate is present in the majority of all phyla, except for Firmicutes, and this synthesis is most prevalent in Bacteroidetes and Fusobacteria. Only a few genomes in the Bacteroidetes phylum contain the role Thi4, which can replace the longer HET-P biosynthesis route. The ThiH role in the longer HET-P synthesis route is most commonly used by HGM organisms; in contrast, the ThiO role is rarely present and is only present in Actinobacteria and Proteobacteria genomes. In total, 56% of the HGM organisms have the ability to produce thiamin.

### Comparison with experimental data

To assess the validity of our predictions, we compared our results with existing experimental data in the literature. Here, we considered only experimental studies that used defined media added specific B-vitamins for growth, or that tested for B-vitamin requirements or secretions specifically. We found experimental evidence of B-vitamin requirements or secretions for 11 of our HGM species. However, for four of these species, experimental data were not found for the specific species that we analyzed. In these cases, we compared the data to our analyzed strains while keeping in mind that strains within a species can differ substantially (Li et al., 2009). In total, we made eight predictions for each of the 16 strains, resulting in 128 predictions. We found that 113 of our predictions matched the experimental data (88%, Table 4). This result suggests that predictions concerning bacterial metabolism can be made based on well-annotated genomes.

**Table 4:**
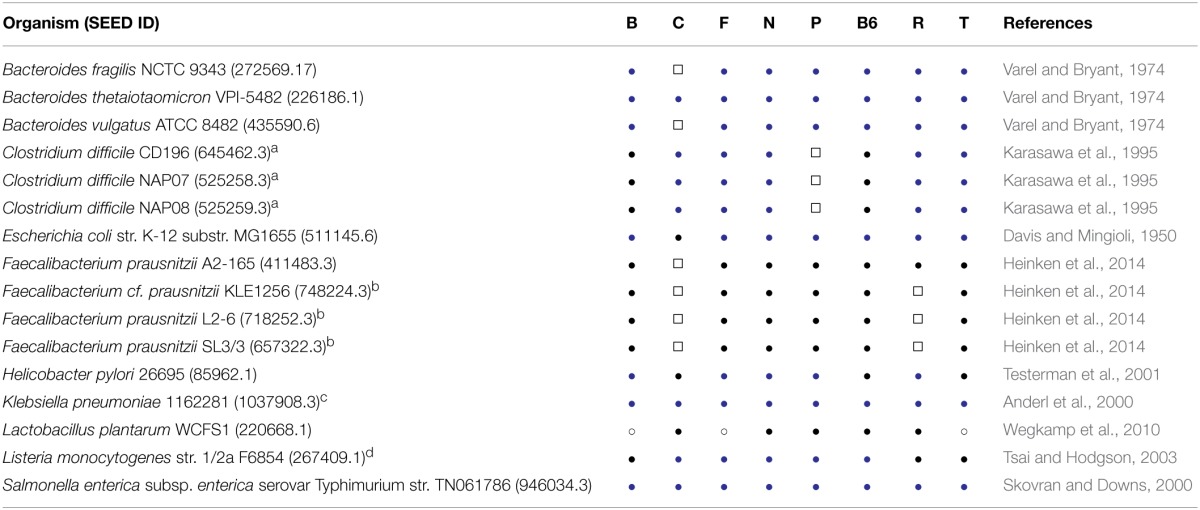
**Comparison of genomic predictions and experimental evidence**.

*B, biotin, C, cobalamin, F, folate, N, niacin, P, pantothenate, B6, pyridoxine, R, riboflavin, T, thiamin. Filled circles represent agreeing predictions and data, empty shapes represent mismatches. 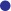, predicted producers, ●, predicted non-producers, □, predicted producers, ◦, predicted non-producers. Footnotes list the comparative strain in the cases where the predicted and experimental strains are different*.

*^a^C. difficile VPI 10463, KZ 1626, KZ 1630, KZ 1647, and KZ 1748*.

*^b^F. prausnitzii A2-165*.

*^c^K. pneumonia Kp1 and Kp102M*.

*^d^L. monocytogenes 10403, EGD-e, and L028*.

### B-vitamin synthesis patterns in HGM and other microbial genomes

**Figure 9:**
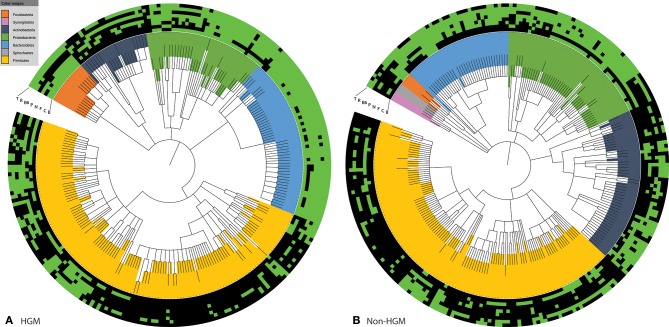
**NCBI based taxonomic trees and the presence or absence of the eight B-vitamin biosynthesis pathways**. The two taxonomic trees show the **(A)** 256 HGM genomes and **(B)** the 257 non-HGM genomes, along with heatmaps showing the presence (green) or absence (black) of each vitamin pathway. The taxonomic trees were produced using PhyloT: a tree generator (http://phylot.biobyte.de/index.html), and visualized through iTOL (http://itol.embl.de/) (Letunic and Bork, 2007, 2011).

Examining the variation of the different synthesis pathways in our former analysis, we wondered how the combinations of the vitamins synthesized varied across the HGM genomes. Our data consist of binary information regarding the distribution of pathways in 256 HGM and 257 non-HGM genomes, i.e., the presence or absence of a vitamin biosynthesis pathway in a genome (Figure 9). We investigated the 2^8^ = 256 possible patterns of the eight studied pathways. Only 68 (27%) of the 256 possible pathway patterns were found in the 256 HGM genomes (Supplementary Table 1), suggesting that the occurrences of these B-vitamin pathways may not be independent.

**Figure 10:**
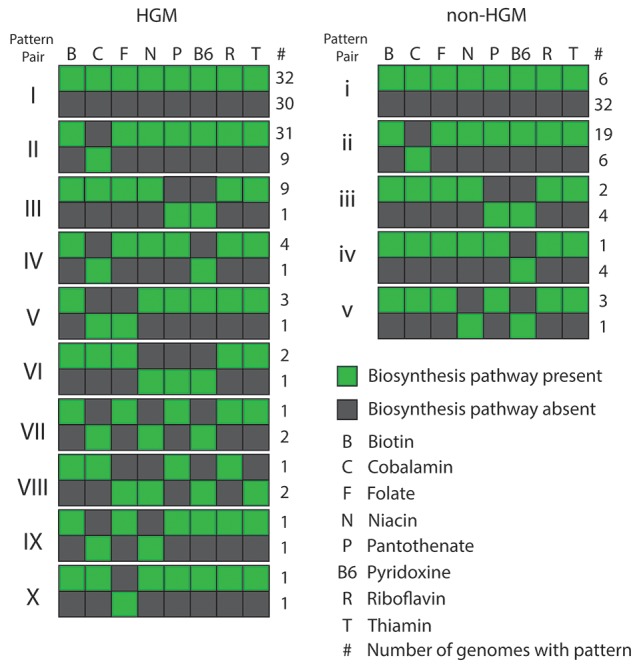
**Inversed pattern pairs of HGM and non-HGM genomes**. The HGM genomes showed ten pairs of inversed patters, whereas the non-HGM contained 5 pairs.

To determine whether the pattern occurrence and distribution was HGM specific or a general feature of bacteria, we inspected the B-vitamin metabolism distribution in 257 bacterial genomes (non-HGM). We identified four groups: (1) 32 HGM-specific patterns, (2) 36 patterns present in both HGM and non-HGM sets, (3) 36 patterns found only in non-HGM genomes, and (4) 152 patterns found in neither. The 32 HGM-specific patterns were present in 68 (27%) of the HGM genomes, and each pattern was found in eight or fewer genomes. Three HGM-specific patterns were found in eight genomes each: (i) the presence of only niacin, pyridoxine, and thiamin pathways was found only in Actinobacteria, (ii) the absence of all pathways except for niacin was found in six Firmicutes and two Actinobacteria genomes, (iii) the presence of all pathways except for biotin and folate biosynthesis was found in five Firmicutes and three Proteobacteria genomes. The remaining HGM-specific patterns were found in four or fewer genomes each. The presence of these HGM-specific vitamin biosynthesis patterns suggests that human gut microbes have evolved to suit gut-specific conditions, resulting in complementary combinations of synthesized B-vitamins. Accordingly, we observed ten pairs of inversed patterns, in which pathways that were present or absent in one genome were absent or present in another, respectively (Figure 10). Such pairs of organisms may be candidates for mutualistic or symbiotic interactions. For example, several Bacteroidetes and Proteobacteria genomes contained all biosynthesis pathways, except for cobalamin synthesis. In the complementary pattern, found in eight Firmicutes genomes, all biosynthesis pathways, except for cobalamin synthesis, were absent. In comparison, only five pairs of inversed patterns were observed in the analyzed non-HGM genomes.

We analyzed the distribution of pathway patterns of the HGM genomes and noted similarities in the eight vitamin biosynthesis capabilities at the phylum, class, order, and family levels. Genomes in the phylum Actinobacteria demonstrated variations in pathway distributions; however, the most conserved pattern, the biosynthesis of niacin, pyridoxine, and thiamin, was present in eight (35%) genomes, and all of these genomes were from the order Bifidobacteriales and the family Bifidobacteriaceae (Figure 9, Supplementary Table 1). Pathway distribution was highly conserved in the phylum Bacteroidetes, which displayed only six different patterns. The two most common patterns in the Bacteroidetes phylum were the biosynthesis of all eight vitamins, which was present in 26 (51%) of the genomes in the phylum, and the synthesis of all vitamins except for cobalamin, which appeared in 17 (33%) of the Bacteroidetes genomes. Three genomes could not produce cobalamin and pyridoxine, and three did not meet the requirements for consideration as cobalamin and folate producers. A single organism in the Bacteroidetes phylum could produce all vitamins except for biotin and cobalamin. One genome of the family Prevotellaceae, *P. salivae* DSM 15606, was missing five biosynthesis pathways, and this organism only produced pantothenate, pyridoxine, and riboflavin. The phylum Firmicutes demonstrates the highest variability in pathway patterns, containing 43 patterns in 130 genomes. However, this variability is not surprising in light of the taxonomic diversity within the phylum itself. The most common pattern was the absence of all biosynthesis pathways and was found in 30 genomes, 26 of which belong to the Lactobacillales order of the Bacilli class. The Lactobacillales order also contained 9 out of 10 Firmicutes genomes that contained only riboflavin biosynthesis. Genomes of the Clostridia class showed an even larger diversity of pathway patterns; these genomes contained 28 out of the 43 Firmicutes patterns. The most common Clostridia pattern, the absence of all synthesis pathways except cobalamin, was found in seven organisms. All the analyzed Fusobacteria genomes belonged to the family Fusobacteriaceae and displayed conserved patterns of pathways. The most common pattern in nine of the 14 genomes was the absence of only the pantothenate and pyridoxine synthesis pathways. The pantothenate biosynthesis pathway was absent in all of the genomes, whereas all genomes contained the biosynthesis pathways for biotin, cobalamin, riboflavin, and thiamin. The phylum Proteobacteria showed surprisingly conserved patterns in light of the high divergence of the taxon. The class Gammaproteobacteria contained 12 out of the 13 Proteobacteria genomes that contained the biosynthesis of all vitamins except for cobalamin. The only other genome to show this pattern was the Betaproteobacteria *Ralstonia* sp. 5_7_47FAA. The remaining 14 patterns were only found in six or fewer genomes in the phylum, and 10 patterns were observed in only a single organism each. Finally, few pathway patterns were shared between the five phyla. The presence of all pathways, except for cobalamin, was observed in Bacteroidetes, Firmicutes, and Proteobacteria in 17, 1, and 13 genomes, respectively. Only nine out of the 68 patterns observed in the HGM genomes were shared between two phyla, whereas 58 patterns appeared in a single phylum each.

Taken together, the distribution of pathway patterns was relatively conserved, with only 68 patterns appearing in the 256 HGM genomes. A third of these patterns (20 out of 68) were the inverse of another pattern, suggesting the existence of symbiotic relationships for B-vitamin metabolism in the gut microbiota.

### Amount of HGM B-vitamins available to the gut

Considering that the B-vitamin biosynthesis pathways seem to be prevalent in the human gut microbiota genomes, we wondered whether gut bacteria have the collective capacity to produce sufficient B-vitamins for daily human requirements. The assumptions underlying the calculation are described in the Method Section. Overall, only four of the eight B-vitamins are predicted to be produced in amounts that could cover at least a quarter of the suggested dietary intake (Table 5). It must be noted that the calculated values are speculative and do not represent the true amount of B-vitamins provided by the human gut microbiota.

**Table 5:** **Estimated maximal percentage of daily reference intake of the eight vitamins that could be provided by the human gut microbiota**.

**Vitamin**	**Intracellular concentration [mmol/gDW]**	**DRI^a^ [mg/day]**	**HGM_ratio_**	**%DRI from HGM**
Biotin	9.0 ^*^ 10^−7^	0.03	0.40	4.5
Cobalamin	8.5 ^*^ 10^−8^	0.0024	0.42	31
Folate^b^	5.0 ^*^ 10^−5^	0.4	0.43	37
Niacin^b^	3.3 ^*^ 10^−3^	15	0.63	27
Pantothenate	2.3 ^*^ 10^−6^	5	0.51	0.078
Pyridoxine^b^	5.8 ^*^ 10^−4^	1.3	0.50	86
Riboflavin	9.0 ^*^ 10^−6^	1.2	0.65	2.8
Thiamin^b^	8.7 ^*^ 10^−6^	1.15	0.56	2.3

a *Dietary reference intakes (Standing Committee on the Scientific Evaluation of Dietary Reference Intakes and Its Panel on Folate, Other B Vitamins, And Choline, 1998). Values averaged for male and female references intakes (ages 19–50)*.

b *Atomic mass for dihydrofolic acid, nicotinic acid, pyridoxine 5'-phosphate, and thiamine monophoshate*.

## Discussion

In this study, we predicted the B-vitamin biosynthesis of 256 known human gut microorganisms based on their genome annotations alone. Our key results are the following: (i) the majority of our genome-based predictions match published experimental data; (ii) B-vitamin biosynthesis is common in the HGM; and (iii) inversed patterns of vitamin synthesis suggest symbiotic relationships among HGM organisms with regard to B-vitamins. Taken together, our data support the idea that the human gut microbiota has co-evolved relationships that are specific to the gut environment.

The predictions of B-vitamin synthesis capability agreed well with experimental data suggesting that the genomic analyses of well-defined pathways can provide much information regarding strain-specific metabolism. The prediction had a 12% error rate, which represents a lower bound for the other HGM capabilities (Table 4) as we compared against well-studied organisms with a higher genome completeness than expected for genomes assembled from metagenomic studies. One example of a wrong prediction is *L. plantarum* WCFS1, in which all necessary functional roles of the biotin pathway were missing. Because biotin is not required for its growth (Wegkamp et al., 2010), either the organism does not need biotin or the manual curation of the pathway requires additional data. The *L. plantarum* WCFS1 genome contains the biotin-dependent protein biotin-protein ligase (Supplementary Table 3), which suggests a need for biotin. All analyzed *C. difficile* genomes were predicted to produce pantothenate as they contained all essential roles except ASPDC. However, pantothenate is required in the growth medium of the comparison strain (Karasawa et al., 1995). The strain difference may explain the discrepancy but it is also possible that our essential role constraints are too loose and that the absence of ASPDC should lead to a negative prediction, even when it is the only missing functional role in the pathway. The four *F. prausnitzii* genomes were missing all essential roles for folate production and were therefore predicted to require folate in their growth media. In contrast, no significant folic acid consumption of *F. prausnitzii* A2-165 has been reported (Heinken et al., 2014) and additionally, it can grow in folic acid free medium (personal communication, Dr. Delphine Saulnier). However, the genome of *F. prausnitzii* A2-165 contains an annotated folate transporter (Heinken et al., 2014) and the folate-dependent methionyl-tRNA formyltransferase (UniProt ID: C7H5H7). We propose that *F. prausnitzii* A2-165 does not require folates for growth, although the presence of folate in the growth medium might enhance the growth rate. *L. plantarum* WCFS1 is missing all PABA functional roles while it has been shown to synthesize folate in the absence of folate precursors and PABA in the growth medium (Wegkamp et al., 2010). This contradiction suggests an unknown route for PABA biosynthesis, which is likely considering that two missing archaeal enzyme families in the folate biosynthesis pathway had recently been discovered (De Crecy-Lagard et al., 2012).

The two most commonly synthesized vitamins of the human gut microbiota genomes were riboflavin and niacin, with 166 and 162 predicted producers, respectively. The gut-microbial production of riboflavin has been associated with the immune response through the activation of T-cells (Kjer-Nielsen et al., 2012). Riboflavin has also been shown to be involved in the extracellular electron transport chain in *F. prausnitzii* (Khan et al., 2012). The conservation of the riboflavin biosynthesis pathway can be explained by the exclusive importance of its derivatives, FAD and FMN, because approximately 1–3% of cellular proteins are flavoproteins (De Colibus and Mattevi, 2006; Abbas and Sibirny, 2011). Niacin, a precursor for NAD, is another essential cofactor. NAD and its reduced and phosphorylated derivatives (NADH, NADP, and NADPH) have many functions in cells, such as serving as hydride donors and acceptors in redox reactions (Belenky et al., 2007; Houtkooper et al., 2010), participation in bacterial and DNA ligase reactions (Wilkinson et al., 2001), and roles as molecules that signal the cellular redox status (Houtkooper et al., 2010). However, the two synthesis pathways are distributed differently over the five phyla. Riboflavin synthesis is mainly found in Bacteroidetes, Proteobacteria, and Fusobacteria, but it is only found in half of the Firmicutes genomes and very few Actinobacteria (Figure 7). In contrast, the niacin biosynthesis pathway is more evenly distributed over the genomes of the five phyla (Figure 4). Such differences between the distributions of these two pathways can have various explanations. First, this variation may reflect their evolutionary history; riboflavin synthesis appears to be more ancient than the NAD biosynthesis pathway (Gazzaniga et al., 2009). Second, the biosynthesis of riboflavin and its derivatives is a quite straightforward pathway (Abbas and Sibirny, 2011), whereas the biosynthesis of NAD is very complex and includes numerous versions of salvage pathways in various bacterial taxa (Kurnasov et al., 2003; Gerdes et al., 2006; Galeazzi et al., 2011; Cialabrini et al., 2013).

The human gut microbiota is a large microbial community, in which metabolites are shared among individual microorganisms (Wolin, 1974; Chassard et al., 2005). The production of all vitamins and the absence of all biosynthesis pathways was one of the most common patterns in the HGM (Figure 10). The absence of all biosynthesis pathways was only found in the Firmicutes phylum, whereas the presence of all eight pathways was found mainly in the Bacteroidetes and in several Proteobacteria (Figure 9, Supplementary Table 2). This distribution of vitamin biosynthesis patterns is interesting given that a higher ratio of Bacteroidetes to Firmicutes has been linked to a healthier gut microbiota compared with that in obese individuals (Turnbaugh et al., 2006). Inversed pattern pairs in HGM was twice as frequent than in non-HGM, and 70% more genomes were associated with these pairs in the HGM compared to non-HGM (Figure 10). These results suggest that the trait of sharing B-vitamins has evolved in the gut microbes.

HGM can supply the host with biotin, folate, and riboflavin (Hill, 1997; Rossi et al., 2011). Human colonocytes contain transporters for several B-vitamins, such as RFT1 for riboflavin (Yonezawa et al., 2008), FOLR1 for folate (Crott et al., 2008), and the multivitamin transporters SMCT1 (Li et al., 2003) and SMVT (Prasad et al., 1999). It has therefore been speculated that the HGM contributes to B-vitamin homeostasis (Said, 2004) because most of the dietary vitamin absorption occurs in the small intestine. The large intestine contains the highest density of microbes in the human gut; thus, it seems likely that bacteria provide human intestinal cells with sufficient B-vitamins to avoid deficiencies during short periods of vitamin-poor diet. According to our estimation, the gut microbiota would not be able to provide the host with the daily recommended intake of B-vitamins (Table 5) and a large portion of these vitamins may be taken up by non-producing gut microbes, which compete with the host. In fact, our results indicate that human gut microbes actively synthesize B-vitamins and provide them to their neighboring bacteria through symbiotic relationships. A high fraction of the recommended pyridoxine intake and about a third of the recommended intake of cobalamin and folate could be available from the gut bacteria (Table 5). Consistently, these vitamins are thought to be produced by the human gut microbiota in addition to dietary uptake (Herbert, 1963; Roth, 1981; Leblanc et al., 2013). In fact, pyridoxine deficiency due to dietary deficiency is rarely observed (Rucker et al., 2007). Cobalamin and folate deficiencies are mainly reported in elderly people (Herbert, 1963; Andres et al., 2004; Clarke et al., 2004), who have been shown to have a less diverse and metabolically active microbiota (Woodmansey et al., 2004; Claesson et al., 2011). In addition, cobalamin and folate have several metabolites derived beyond their basic forms, and the host may not absorb these compounds. For example, it has been shown that little of the corrinoids found in human feces are gut-bacteria derived (Allen and Stabler, 2008), and it has been proposed that the most of the corrinoids produced by the gut bacteria are taken up by non-producers in the gut (Degnan et al., 2014). Vitamin B12 has recently been suggested as a modulator of gut microbial ecology (Degnan et al., 2014). We propose that other B-vitamins could also have a gut microbiota-modulating role. In this study, we have not analyzed vitamin transporters because little is known about the B-vitamin export from bacterial cells (Rodionova et al., 2014). However, if B-vitamins are released into the gut during cell lysis, any vitamin producer can be considered a vitamin source, regardless of its transporter expression. Taken together, our results highlight the fact that the microbiota does indeed contribute to the B-vitamin pool of the gut and that the host can benefit to some extent from the B-vitamin biosynthesis of the microbiota.

In conclusion, our genomic assessment of 256 HGM organisms suggests that these organisms have co-evolved in the gut and shows several possible symbiotic and competitive relations within the gut microbiota. The co-evolution of B-vitamin biosynthesis in gut microorganisms has likely benefitted the host's vitamin homeostasis.

### Conflict of interest statement

The authors declare that the research was conducted in the absence of any commercial or financial relationships that could be construed as a potential conflict of interest.
