# Supplementary material for: Systematic genome assessment of B-vitamin biosynthesis suggests co-operation among gut microbes
Source: Front Genet. 2015 Apr 20;6:148. doi: 10.3389/fgene.2015.00148 (PMC4403557; doi:10.3389/fgene.2015.00148)
Supplement: Supplementary file 3 [file Supplementaldataoverview.DOCX]

***Supplementary Material***

**Systematic genome assessment of B-vitamin biosynthesis suggests co-operation among gut microbes**

**Stefanía Magnúsdóttir^1^, Dmitry Ravcheev^1^, Valérie de Crécy-Lagard^2^, Ines Thiele^1,*^**

^1^Luxembourg Centre for Systems Biomedicine, University of Luxembourg, Esch-sur-Alzette, Luxembourg
^2^Department of Microbiology and Cell Science and Genetics Institute, University of Florida, Gainesville, FL, USA

*** Correspondence:** : Ines Thiele, Luxembourg Centre for Systems Biomedicine, University of Luxembourg, 7, Avenue des Hauts-Fourneaux, Luxembourg.

1. **Supplementary Data**

The file “PEG.fasta” contains amino acid sequences of all protein encoding genes (PEGs) that appear in the eight subsystems for the 256 human gut microbiota (HGM) genomes.

1. **Supplementary Figures and Tables**

All supplementary tables can be found in the file “SupplementaryTables.xlsx”. Supplementary Figures 1 and 2 can be found in the files “SupplementaryFigure_01.png” and “SupplementaryFigure_02.png”.

Below is the list of supplementary materials provided in the two files and the corresponding table and figure legends.

## Supplementary Tables

**Supplementary Table 1. B-vitamin pathways present and absent in the studied genomes.** Presence (1) and absence (0) of the eight B-vitamin biosynthesis pathways in the 256 HGM and 257 non-HGM genomes.

**Supplementary Table 2. Functional role abbreviations and full names of the eight B-vitamin subsystems.** All PubSEED functional roles associated with each abbreviation used in Figures 1-8 and Table 2.

**Supplementary Table 3. Biotin biosynthesis subsystem, HGM genomes.** PEG numbers of each functional role found in the subsystem for the 256 HGM genomes.

**Supplementary Table 4.** **Cobalamin biosynthesis subsystem, HGM genomes.** PEG numbers of each functional role found in the subsystem for the 256 HGM genomes.

**Supplementary Table 5.** **Folate biosynthesis subsystem, HGM genomes.** PEG numbers of each functional role found in the subsystem for the 256 HGM genomes.

**Supplementary Table 6.** **Niacin biosynthesis subsystem, HGM genomes.** PEG numbers of each functional role found in the subsystem for the 256 HGM genomes.

**Supplementary Table 7.** **Pantothenate biosynthesis subsystem, HGM genomes.** PEG numbers of each functional role found in the subsystem for the 256 HGM genomes.

**Supplementary Table 8.** **Pyridoxine biosynthesis subsystem, HGM genomes.** PEG numbers of each functional role found in the subsystem for the 256 HGM genomes.

**Supplementary Table 9.** **Riboflavin biosynthesis subsystem, HGM genomes.** PEG numbers of each functional role found in the subsystem for the 256 HGM genomes.

**Supplementary Table 10.** **Thiamin biosynthesis subsystem, HGM genomes.** PEG numbers of each functional role found in the subsystem for the 256 HGM genomes.

**Supplementary Table 11.** **Biotin biosynthesis subsystem, non-HGM genomes.** PEG numbers of each functional role found in the subsystem for the 257 non-HGM genomes.

**Supplementary Table 12.** **Cobalamin biosynthesis subsystem, non-HGM genomes.** PEG numbers of each functional role found in the subsystem for the 257 non-HGM genomes.

**Supplementary Table 13.** **Folate biosynthesis subsystem, non-HGM genomes.** PEG numbers of each functional role found in the subsystem for the 257 non-HGM genomes.

**Supplementary Table 14.** **Niacin biosynthesis subsystem, non-HGM genomes.** PEG numbers of each functional role found in the subsystem for the 257 non-HGM genomes.

**Supplementary Table 15.** **Pantothenate biosynthesis subsystem, non-HGM genomes.** PEG numbers of each functional role found in the subsystem for the 257 non-HGM genomes.

**Supplementary Table 16.** **Pyridoxine biosynthesis subsystem, non-HGM genomes.** PEG numbers of each functional role found in the subsystem for the 257 non-HGM genomes.

**Supplementary Table 17.** **Riboflavin biosynthesis subsystem, non-HGM genomes.** PEG numbers of each functional role found in the subsystem for the 257 non-HGM genomes.

**Supplementary Table 18.** **Thiamin biosynthesis subsystem, non-HGM genomes.** PEG numbers of each functional role found in the subsystem for the 257 non-HGM genomes.

## Supplementary Figures

**Supplementary Figure 1.** **Normal** **NCBI based taxonomic tree of the 256 HGM genomes and the presence or absence of the eight B-vitamin biosynthesis pathways.** In the heatmap, green shows the presence and black represents the absence of a pathway. The taxonomic tree was produced using PhyloT: a tree generator (<http://phylot.biobyte.de/index.html>), and visualized through iTOL (<http://itol.embl.de/>) (Letunic and Bork, 2007; 2011).

**Supplementary Figure 2.** **Normal** **NCBI based taxonomic tree of the 257 non-HGM genomes and the presence or absence of the eight B-vitamin biosynthesis pathways.** In the heatmap, green shows the presence and black represents the absence of a pathway. The taxonomic tree was produced using PhyloT: a tree generator (<http://phylot.biobyte.de/index.html>), and visualized through iTOL (<http://itol.embl.de/>) (Letunic and Bork, 2007; 2011).

## References

Letunic, I., and Bork, P. (2007). Interactive Tree Of Life (iTOL): an online tool for phylogenetic tree display and annotation. *Bioinformatics* 23**,** 127-128. doi: 10.1093/bioinformatics/btl529.

Letunic, I., and Bork, P. (2011). Interactive Tree Of Life v2: online annotation and display of phylogenetic trees made easy. *Nucleic Acids Research* 39**,** W475-W478. doi: 10.1093/nar/gkr201.
